# Supplementary material for: Co-Inoculation with Azospirillum brasilense and Bradyrhizobium sp. Enhances Nitrogen Uptake and Yield in Field-Grown Cowpea and Did Not Change N-Fertilizer Recovery
Source: Plants (Basel). 2022 Jul 14;11(14):1847. doi: 10.3390/plants11141847 (PMC9321259; doi:10.3390/plants11141847)
Supplement: Supplementary file 1 [file plants-11-01847-s001.zip › plants-1792512-supplementary.pdf]

Journal: Plants

Co-inoculation with *Azospirillum brasilense* and *Bradyrhizobium* sp. enhances nitrogen uptake and yield in field-grown cowpea and did not change N-fertilizer recovery

Figure S1. Daily rainfall (bar) and temperature (lines) during cowpea cropping season.

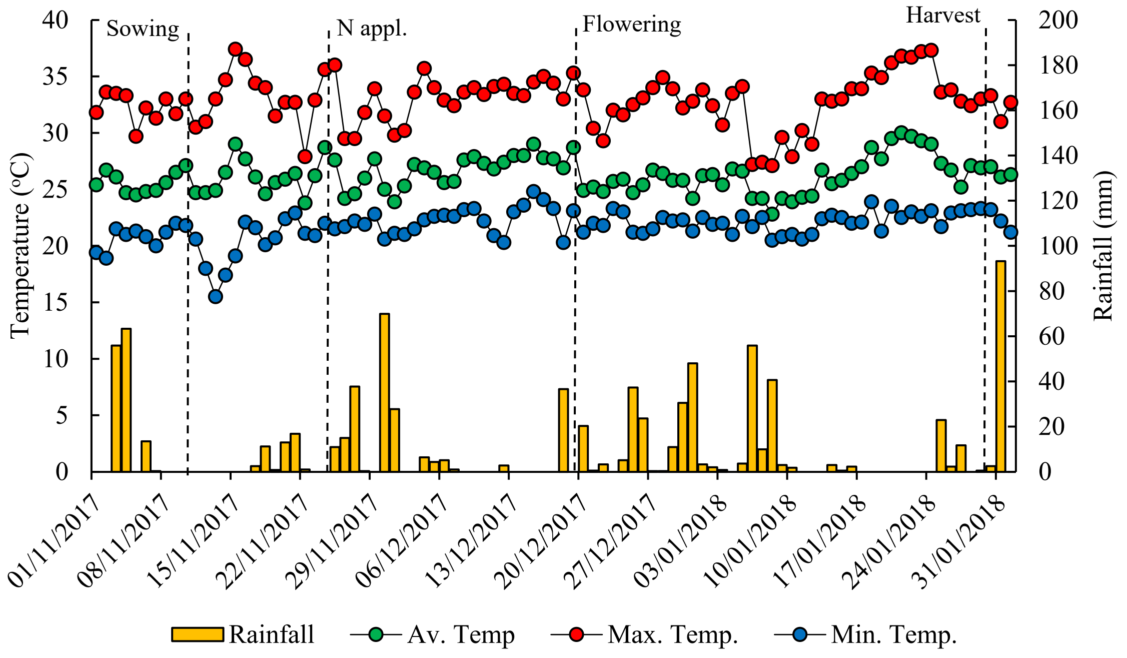

Av. Temp., Max. Temp. and Min. Temp. refers to average, maximum and minimum temperatures, respectively.

Table S1. Bradyrhizobia populations and *Azospirillum* sp. most probably number (MPN) in bulk soil surrounding the roots at cowpea full flowering.

**Bradyrhizobia populations**	**MPN (cells g^{-1} soil)**
Single *Bradyrhizobium* sp. inoculation	10.1×10^6
Co-inoculation with *Bradyrhizobium* sp. + *A. brasilense*	12.4×10^6
***Azospirillum* sp.**	**MPN (cells g^{-1} soil)**
Single *Bradyrhizobium* sp. inoculation	4.3×10^4
Co-inoculation with *Bradyrhizobium* sp. + *A. brasilense*	6.8×10^6

Number of repetitions (n) = 10.
